# Characterization of *Cistus* × *incanus* L. and *Cistus ladanifer* L. Extracts as Potential Multifunctional Antioxidant Ingredients for Skin Protecting Cosmetics

**DOI:** 10.3390/antiox9030202

**Published:** 2020-03-01

**Authors:** Katarzyna Gaweł-Bęben, Wirginia Kukula-Koch, Uliana Hoian, Marcin Czop, Marcelina Strzępek-Gomółka, Beata Antosiewicz

**Affiliations:** 1 Department of Cosmetology, University of Information Technology and Management in Rzeszów, Sucharskiego 2, 35-225 Rzeszów, Poland; uhoian@wsiz.rzeszow.pl (U.H.); mstrzepek@wsiz.rzeszow.pl (M.S.-G.); bantosiewicz@wsiz.rzeszow.pl (B.A.); 2 Department of Pharmacognosy, Medical University of Lublin, Chodźki 1, 20-093 Lublin, Poland; virginia.kukula@gmail.com; 3 Department of Clinical Genetics, Medical University of Lublin, Radziwiłłowska 11, 20-080 Lublin, Poland; marcin.czop@umLub.pl

**Keywords:** *Cistus incanus*, *Cistus ladanifer*, antioxidant, tyrosinase, melanoma, in vitro cytotoxicity, sun protection factor (SPF)

## Abstract

Skin is constantly exposed to harmful environmental factors, causing photo-oxidative stress in cells and leading to the development of health and aesthetic problems. Multifunctional ingredients of everyday skincare products, possessing antioxidant, UV-protecting, anti-hyperpigmentation, and skin cancer-preventing properties are in high demand. Due to the high content of polyphenolic compounds *Cistus* × *incanus* L. and *Cistus ladanifer* L. are potentially interesting sources of cosmetic ingredients with multiple skin protecting functions. In this study eight extracts from dried *C. incanus* and *C. ladanifer*—aerial parts were prepared using 60% (*v*/*v*) or 100% (*v*/*v*) methanol, on a magnetic stirrer or in Soxhlet apparatus, and compared for their content of phytochemicals and properties important for the skin protection. Extracts from *C. incanus* prepared in 60% (*v*/*v*) methanol contained the highest amount of polyphenolic compounds (331.82–347.27 mg GAE/g DW) and showed the most significant antioxidant activity (IC_50_ = 3.81–4.05 µg/mL). *C. incanus* extracts were also effective tyrosinase inhibitors (30–70% inhibition at 100 µg/mL). Statistical correlation analysis revealed that epicatechin, epigallocatechin gallate (EGCG), and myricitrin may be responsible for the antioxidant and tyrosinase inhibitory potential of *C. incanus* extracts. All analyzed extracts were cytotoxic for human melanoma cells A375 (IC_50_ = 57.80–199.01 µg/mL), with *C. incanus* extract prepared in 100% (*v*/*v*) methanol using Soxhlet extraction being the most effective. The extracts did not significantly impair the growth of noncancerous human keratinocytes HaCaT. *C. incanus* and C*. ladanifer* extracts possess also natural sun protecting activity (SPF 3.42–3.77 at 100 µg/mL), enhancing their anti-hyperpigmentation and anti-melanoma potential.

## 1. Introduction

Skin is the largest organ of the human body that is constantly exposed to various environmental factors with potential harmful effect—high or low temperatures, air pollution, UV radiation. All of these factors may cause damage to the skin components, causing oxidative stress, and increasing the risk of pigmentation disorders and skin cancer [1]. As is it not possible to remove most of the stressing factors from the environment, a healthy diet rich in natural antioxidants and regular application of cosmetics with photoprotecting, antioxidant, and skin calming properties seem to be the key aspects in the chemoprevention of skin disorders [2,3]. The most promising active ingredients of these type of cosmetic products are plant extracts, rich in polyphenolic compounds displaying multiple protecting functions. Particularly interesting for this type of application are extracts obtained from plants native to the Mediterranean region. Due to the constant exposure to stressful environmental conditions, including water deficiency, high temperature, and intensive solar radiation these plants contain broad spectrum of polyphenolic compounds protecting them from the negative consequences of the photo-oxidative stress. Polyphenolic compounds, especially flavonoids, are known for their ability to scavenge reactive oxygen species (ROS), chelate transition metal ions, and reduce lipid peroxidation. These compounds were also shown to possess skin calming, UV-protecting, lightening and skin cancer preventing properties, confirmed in many in vitro and in vivo studies [4,5,6].

Mediterranean shrub species *Cistus incanus* L. and *Cistus ladanifer* L. are naturally rich in polyphenolic compounds and thus representing a potential source of bioactive ingredients for skin protecting cosmetics. *Cistus* sp. are rich in flavonoids, particularly from the group of flavonols (quercetin, kaempferol, and myricetin) and flavan-3-ols (catechins, gallocatechins, proanthocyanidins). Additional phytochemicals found in *Cistus* aerial parts include terpenes, fatty acids, phytohormones, and vitamins [7]. Extracts and active compounds isolated from *C. incanus* and *C. ladanifer* were shown to possess several properties valuable for skin protecting applications. *C. incanus* infusions were used in traditional medicine to treat various skin disorders due to their anti-inflammatory potential. Extracts from *C. incanus* leaves showed antimycotic, antibacterial, and antiviral properties in vitro [8,9,10,11]. Aqueous and ethanolic extracts of *C. incanus* possess also significant antioxidant activity [12]. *C. ladanifer* extracts showed antimicrobial activity against several Gram-positive and Gram-negative bacteria strains, pathogenic yeast, and fungi [13,14,15]. Aqueous extracts from this plant possess also anti-inflammatory and anti-nociceptive activities in vivo [16]. Several biological properties of *Cistus* extracts was correlated with the high content of polyphenolic compounds. Cosmetic application of *Cistus* sp. is also related to the labdanum, a resin obtained from *C. ladanifer* has an excellent odor and is, therefore, used in the manufacture of perfumes, cosmetics, soaps, detergents, and deodorants [17]. Based on the literature data, extracts from *C. incanus* and *C. ladanifer*—rich in polyphenolic compounds—seem to be a very interesting active ingredients of cosmetic formulations, protecting the skin cells from oxidative stress, skin cancer, and other harmful effects of UV radiation.

The conventional methods of polyphenol recovery from the plant are based on the solid–liquid solvent extraction, most often coupled with the use of heat or agitation. It is generally known that the yield of extracted polyphenols depends on the chemical composition and physical characteristics of the plant material as well as on the type of solvent used, their polarity, extraction manner, contact time, and temperature. The results can vary even by one order of magnitude when different procedures are used for the same sample [18]. Among techniques used to extract active constituents from plant material the most commonly used are Soxhlet extraction and turboextraction using a magnetic stirrer. The main advantage of the Soxhlet extraction is the displacement of transfer equilibrium by repeatedly bringing fresh solvent into contact with the plant material. However, the main disadvantage of this method is the possibility of thermal degradation of the isolated phytochemicals as the extraction usually occurs at the boiling point of the solvent [19].

In the presented study, the authors aimed to optimize the extraction conditions of *C. incanus* and *C. ladanifer* dried aerial parts in order to obtain the extracts containing high amounts of various polyphenolic compounds. Prepared extracts were also compared for their biological properties important for the protection of skin from the harmful effects of long time UV exposure: antioxidant activity, anti-cancer properties against human melanoma and squamous cell carcinoma cells, tyrosinase inhibitory activity and in vitro sun protection factor (SPF). Finally, biological properties of the extracts were correlated with the content of particular polyphenolic compounds in order to emphasize the potential application of *C. incanus* and *C. ladanifer* polyphenolics in the skin protecting cosmetics.

## 2. Materials and Methods 

### 2.1. Chemicals, Reagents, and Cell Lines

A375 (ATCC CRL-1619) human malignant melanoma and human squamous cell carcinoma SCC-15 (ATCC CRL-1623) cell lines were purchased from LGC Standards (Łomianki, Poland). HaCaT immortalized human keratinocytes were purchased from CLS Cell Lines Service GmbH (Eppelheim, Germany). Fetal bovine serum (FBS) was obtained from Pan-Biotech (Aidenbach, Germany). Dulbecco’s modified Eagle’s medium (DMEM)/high glucose, Dulbecco’s phosphate buffered saline (DPBS), mushroom tyrosinase from *Agaricus bisporus*, 3,4-Dihydroxy-l-phenylalanine (l-DOPA), 2,2-Diphenyl-1-picrylhydrazyl (DPPH), neutral red solution (3.3 g/L), gallic acid, rutoside, quercetin, kojic acid, and DMSO were purchased from Sigma-Aldrich (St. Louis, MO, USA). The purity of the reference compounds exceeded 95%. Acetonitrile, water, and formic acid for LC-MS analyses were purchased from Merck (Darmstadt, Germany). All other reagents were purchased from Honeywell (Warszawa, Poland). All solutions were prepared with ultrapure water (MilliQ Integral II, Merck, Darmstadt, Germany). 

### 2.2. Plant Material and Extraction Procedure

Dried aerial parts of *C. incanus* and *C. ladanifer* from the EU-certified organic farming were purchased from Look Food sp. z o. o., Warszawa and Batom.pl Jozef Lesniak, Krakow, Poland, respectively. The plant material was authenticated by professor in pharmacognosy, prof. Kazimierz Glowniak. A voucher specimen of each plant is being kept in the Department of Cosmetology, The University of Information Technology and Management in Rzeszow, Poland with the appropriate identification numbers: KGB2020_1 (*C. incanus*) and KGB2020_2 (*C. ladanifer*). Three grams of dried *C. inacanus* and *C. ladanifer* were extracted in 300 mL of 60% (*v*/*v*) methanol:water solution or 100% methanol for 16 h on a magnetic stirrer (60% M and 100% M) or using Soxhlet apparatus (60% SOX and 100% SOX). Obtained extracts were filtered through Whatman paper and 0.45 µm syringe filter followed by solvent evaporation at 37 °C. Dried extracts were stored at 4 °C until analysis.

### 2.3. Determination of Total Phenolic Compounds

The content of total phenolic compounds was determined as described by Fukumoto and Mazza [20] with slight modifications. Briefly, 150 µL of dissolved extract (100 µg/mL) was mixed with 750 µL Folin–Ciocalteu reagent (1:10 *v*/*v*, in water) and incubated for 5 min at RT. Following addition of 600 µL 7.5% (*m*/*v*) Na_2_CO_3_ the samples were incubated further 30 min at RT in darkness. The absorbance was measured at λ = 740 nm using DR 600 Spectrophotometer (Hach Lange, Wrocław, Poland). The calibration curves were prepared using 0–100 µg/mL gallic acid in 100% (*y* = 0.0102*x* + 0.02; R^2^ = 0.9982) or 60% (*y* = 0.011*x* + 0.004; R^2^ = 0.9929) methanol. The content of phenolic compounds is expressed as gallic acid equivalents (GAE) in mg per g of dried extract weight (DW).

### 2.4. Determination of Flavonoids

The concentration of flavonoids in *Cistus* extracts was measured according to Matejić et al. protocol [21] with some modifications. Briefly, 150 µL of dissolved extracts (1mg/mL) were mixed with 650 µL reaction mixture (61.5 mL 80% C_2_H_5_OH + 1.5 mL 10% Al(NO_3_)_3_·9H_2_O + 1.5 mL 1 M CH_3_COOK). The absorbance of the samples was measured at λ = 415 nm following 40 min incubation at RT in darkness. The calibration curves were prepared using 0–100 µg/mL quercetin in 100% (*y* = 0.0125*x* + 0.0039; R^2^ = 0.9995) or 60% (*y* = 0.0123*x* + 0.0028; R^2^ = 0.9996) methanol. The content of flavonoids is expressed as quercetin equivalents (QuE) per gram of dried extract weight (DW).

### 2.5. LC-MS Analysis

An LC-ESI-Q-TOF-MS based both qualitative and quantitative analysis of *Cistus spp.* extracts was achieved in a tailored method run on an Agilent G3250AA LC/MSD TOF system equipped with an HP 1200 chromatograph and an ESI- Q-TOF-MS spectrometer (Agilent Technologies, Santa Clara, CA, USA). The set was equipped in a degasser (G1322A), a thermostated column chamber, an autosampler (G1329B), a PDA detector (G1315D), and a binary pump (G1312C). The analyses were performed in a gradient method on a Zorbax RP 18 (150 × 2.1 mm, dp = 3.5 µm) HPLC column that included two solvents: water (solvent A) and acetonitrile (solvent B)—both with the addition of 0.1 % of formic acid to enhance the ionization of metabolites. The following composition of gradient was applied: 0 min 2% of B in A, 15 min 25% of B in A, 30 min 45% of B in A, 40 min 95% of B in A, 43 min 2% of B in A. The injection volume of all samples at a concentration of 10 mg/mL was 10 µL, the flow rate of 0.2 mL/min, the analysis run: 55 min and the post run: 10 min. The following UV wavelengths were recorded: 210, 254, 280, 320, and 365 nm. Mass spectrometer was operated in the *m/z* range of 40–1500 u, with a scan rate of 1 spectrum per second, with 110 V of fragmentation energy, 10, 20, and 30 V of fixed collision energies, 350 and 400 °C of gas and sheath gas temperatures, 12 L/min of both gases flow, 35 psig of nebulizer pressure, 4000 V of capillary voltage, and 1000 V of nozzle voltage. The Agilent MassHunter Qualitative Analysis Navigator version: B.08.00 was used to handle the obtained data.

The quantitative evaluation of the molecules of interest was performed based on the calibration curves of several compounds: epicatechin and epigallocatechin gallate for the quantification of catechins, rutoside—for the flavonoid glucosides, gallic acid, caffeic acid for the phenolic acids, and quercetin—for flavonoids aglycones. All calibration curves were prepared out of 6 solutions prepared through dissolving of the stock solution of 1 mg/mL to 0.5, 0.25, 0.1, 0.075, 0.05, and 0.025 mg/mL to include the range of content of the individual metabolites in the extracts. The R^2^ value of all of them exceeded 0.993.

### 2.6. DPPH Radical Scavenging Assay

DPPH radical scavenging was performed as described by Brand-Williams et al. [22]. Diluted extracts (0–500 µg/mL) were mixed in 1:1 ratio with 25 mM DPPH solution in methanol and incubated for 30 min at room temperature in darkness. Absorbance of the samples was measured at λ = 517 nm on DR600 UV-Vis Spectrophotometer (Hach Lange, Wrocław, Poland) using methanol as a blank sample. The percentage of DPPH scavenging activity was calculated for each sample based on the equation

DPPH scavenging activity [%] = [1 − (As/Ac)] × 100%(1)

where: As—absorbance of the sample; Ac—absorbance of the control sample (DPPH + solvent). The IC_50_ value was defined as the concentration of dried extract in µg/mL that is required to scavenge 50% of DPPH radical activity.

### 2.7. In Vitro Cytotoxicity Assay

A375, SCC-15 and HaCaT cell lines were maintained in DMEM supplemented with 10% FBS at 37 °C in a humidified atmosphere with 5% CO_2_. Dried extracts of *C. incanus* and *C. ladanifer* were dissolved in DMSO to final concentration of 50 µg/mL and used to determine in vitro cytotoxicity by neutral red uptake test [23]. The cells (3000 per well) were plated onto a 96-well plate and grown overnight. The cells were then treated with various concentrations of extracts (0–1000 µg/mL) or equal volume of DMSO as a solvent control. Following 48 h of culture the cells were incubated for 3 h in culture medium containing 33 µg/mL neutral red. The cells were rinsed with DPBS and lysed using acidified ethanol solution (50% *v*/*v* ethanol, 1% *v*/*v* acetic acid). The absorbance of the released neutral red was measure using FilterMax F5 microplate reader (Molecular Devices, San Jose, CA, USA) at λ = 540 nm and corrected by the absorbance at λ = 620 nm. Mean absorbance value of the lysate from untreated cells was set as 100% cellular viability and used to calculate the percentage of viable cells following extracts treatment. Obtained results for samples analyzed in a concentration range from 1000 to 3.9 µg/mL were transformed (*x* = log(*x*)) and used to calculate the half minimal inhibitory concentration (IC_50_) using GraphPad Prism 5.0 software (San Diego, CA, USA).

### 2.8. Mushroom Tyrosinase Inhibitory Assay

Determination of the tyrosinase inhibitory properties of *Cistus* extracts was performed according to the protocol described by Studzińska-Sroka and co-workers [24]. Briefly, 675 µL phosphate buffer (100 mM, pH 6.8) was mixed with 375 µL tyrosinase (100 U/mL) and 300 µL of analysed extract or kojic acid in the concentration range from 25–200 µg/mL. Following 10 min incubation at RT in darkness 150 µL L-DOPA (4 mM) was added to each sample and the absorbance of the reaction product (dopaquinone) was measured at λ = 475 nm after another 20 min of incubation. The obtained values were corrected by the absorbance value of the appropriately diluted extract without tyrosinase and L-DOPA. The absorbance of the control sample, containing buffer, tyrosinase, and L-DOPA was set to 100% tyrosinase activity and used to calculate the activity of tyrosinase in other experimental conditions. 

### 2.9. Determination of the Sun Protection Factor (SPF)

Sun protection factor (SPF) was determined in vitro by measuring the absorbance of 100 µg/mL *Cistus* extracts within the wavelength range from 290–320 nm. The solvents (60% (*v*/*v*) methanol:water solution or 100% methanol) were used as blank samples. For SPF calculations the Mansur Equation (2) [25] was applied and EE × I values determined by Sayre [26] (Table 1). were used.

(2) $$\;\mathrm{SPF}=CF\times \overset{320}{\underset{290}{\sum }}\mathrm{EE}\;\left(\lambda \right)\times \;I\;\left(\lambda \right)\times \;\mathrm{Abs}\;\left(\lambda \right)$$

where: EE (λ)—erythemal effect spectrum; I (λ)—solar intensity spectrum; Abs (λ)—absorbance of the sample; CF—correction factor (=10). Abs values of *C. incanus* and *C. ladanifer* extracts diluted in 60% or 100% methanol to 100 µg/mL were determined using DR600 UV-Vis spectrophotometer (Hach Lange, Wrocław, Poland).

### 2.10. Statistical Analysis

All experiments were conducted in at least three replicates. Obtained data were analyzed using GraphPad Prism 7.0 Software (GraphPad Software, San Diego, CA, USA) and Statistica 13.0 Software (StatSoft, Krakow, Poland). The statistical significance between results obtained for different extracts were analyzed using three-way ANOVA (with three qualitative factors) followed by Tukey’s test. Pearson’s correlation analysis was used to check the relationship between the studied variables. Cluster analysis was used to determine the relationship between the tested extracts. All data are showed as Mean ± SD. 

## 3. Results and Discussion

### 3.1. Content of Total Phenolics, Flavonoids, and Antioxidant Activity of *C. incanus* and *C. ladanifer* Extracts

The extracts from dried *C. incanus* and *C. ladanifer* aerial parts prepared using different solvents and procedures were compared for their content of total phenolic compounds and flavonoids (Table 2). The extracts from both *Cistus* species prepared in 60% methanol contained higher amounts of phenolics and flavonoids. Extraction using Soxhlet apparatus slightly increased the content of total phenolic compounds, whereas the content of flavonoids was comparable for both solvents used.

High content of polyphenolic compounds in plant extracts is often correlated with significant antioxidant activity due to the proven reactive oxygen species (ROS) scavenging potential of several polyphenols [27]. In the present study, the strongest antioxidant activity was detected in the extracts containing high amounts of polyphenolic compounds, whereas the extracts containing low levels of phenolics showed lower DPPH scavenging potential. The highest antioxidant activity was shown for 60% SOX (IC_50_ = 3.81 ± 0.36 µg/mL) and 60% M *C. incanus* extract (IC_50_ = 4.05 ± 0.43 µg/mL) (Table 2). Previous studies showed that *C. incanus* extracts and polyphenolic rich fractions are effective DPPH scavengers (IC_50_ = 2.99 ± 1.18 µM for crude ethanolic extract and 0.92 ± 0.10 µM for ethyl acetate fraction in DPPH scavenging assay) [12]. The antioxidant activity of polyphenols from *C. incanus* was also confirmed by in vitro studies, where incubation of hamster fibroblast cells (V79) with *C. incanus* fraction enriched in catechins and procyanidins reduced the intracellular ROS levels by 30–40% [28]. Extracts from *C. ladanifer* were also shown to display significant antioxidant activity that was connected with high content of flavonoids and phenolic acids [29,30,31,32]. Increased ROS levels cause DNA, lipid, and protein damage and aberrant cellular signaling. These aberrations lead to various pathological conditions, including premature skin aging, carcinogenesis and hyperpigmentation disorders [33,34,35]. Phenolic compounds are protective agents in oxidative stress conditions [27]. Regular supplementation of *C. incanus* herbal tea also significantly reduced oxidative stress markers in human volunteers [36]. Thus, it could be expected that topical application of *Cistus* extracts, rich in polyphenols might have beneficial effects for the skin.

### 3.2. Qualitative and Quantitative Analyses of *C. incanus* and *C. ladanifer* Extracts

The differences in the antioxidant potential of analyzed *C. incanus* and *C. ladanifer* extracts are reflected in their phytochemical composition, determined by LC/MS analysis. A careful qualitative analysis of the studied extracts provided the identification of more than twenty major metabolites that belong to the group of polyphenols (Table 3). Below the tentatively identified components are presented together with one of the obtained mass chromatograms showing the rich composition of the extract in the applied conditions. Table 4 presents quantitative analysis of the major compounds identified in prepared extracts.

Having read several scientific publications on *Cistus spp.* and comparing them with the herein presented results it can be clearly seen, that similar components of phenolic nature are identified and listed by different authors [7,12].

In our studies *C. incanus* extracts were generally more rich in phytochemicals from *C. ladanifer* extracts. They contained higher concentration and a broader spectrum of metabolites. Major constituents of *C. incanus* found in the LC-MS analysis were myricetin and its derivatives (myricitrin, myricetin, myricitrine pentoside) and catechin derivatives (epigallocatechin, gallocatechin, epicatechin). The extracts from *C. ladanifer* contained mostly kaempferol glycosides and phenolic acids (gallic and ellagic acids) with a noticeably lower concentration of flavanol derivatives. In case of *C. incanus* samples we found no major differences in the qualitative composition of extracts in comparison with the former studies, whereas the composition of *C. ladanifer* extracts varied slightly from the previous communications. Tomas-Menor and co-investigators [15] in their scientific results also noted lack of catechin derivatives (epicatechin, catechin, or epigallocatechin) in the extracts of *C. ladanifer*. In our studies, among flavanols, only epigallocatechin dimer was identified in this species. Similarly to these studies, we have also tentatively identified uralenneoside and betuloside in *C. ladanifer* extracts, that were not present in *C. incanus* samples. On the other hand, contrary to Tomas-Menor and co-workers, the herein described extracts of *C. ladanifer* were rich in quercetin glycoside and myricetin hexoside, and also contained a small amount of quercetine rutinoside. These differences may be influenced by a different area of origin or various growing conditions of the tested samples.

### 3.3. Tyrosinase Inhibitory Properties of Cistus Extracts

**Figure 1:**
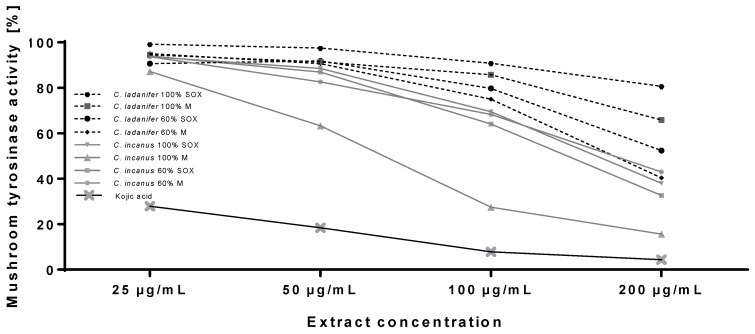
Inhibition of mushroom tyrosinase by *C. incanus* and *C. ladanifer* extracts in comparison with kojic acid; data on graphs represent mean values (*n* = 3).

*Cistus* extracts were then compared for their tyrosinase inhibitory activity. Tyrosinase (EC 1.14.18.1) is a cooper containing enzyme catalyzing the rate limiting conversion of l-tyrosine to l-dihydroxyphenylalanine (l-DOPA) and subsequently to dopaquinone. Due to the key role in melanin synthesis, tyrosinase is the most common target of skin lightening cosmetics used to reduce hyperpigmentation [39]. These types of aesthetic disorders, defined as local accumulation of melanin pigment, are caused by mainly by excessive UV exposure and thus are one of the most important targets of skin protecting cosmetics [40]. As shown in Figure 1, extracts from both *Cistus* species showed significant tyrosinase inhibitory activitiy. The extracts from *C. incanus* were more potent tyrosinase inhibitors than *C. ladanifer* extracts. *C. incanus* extracts at maximum tested concentration (200 µg/mL) showed 55–80% tyrosinase inhibition and around 30% inhibition at the concentration of 100 µg/mL. The most active extract from *C. incanus* (100% M) at 100 µg/mL showed comparable activity with 25 µg/mL kojic acid, a well-known tyrosinase inhibitor commonly used in skin lightening cosmetics. Among *C. ladanifer* extracts, the highest tyrosinase inhibitory activity was detected in 60% M extract that inhibited tyrosinase by 55% at 100 µg/mL. 

The influence of *Cistus* extracts on tyrosinase activity has not been described in the scientific literature to date. However, several compounds identified in this and previous studies in *C. incanus* and *C. ladanifer* extracts were previously shown to act as tyrosinase inhibitors and decrease melanin synthesis in vitro. For example, (−)-epicatechin 3-*O*-gallate (ECG), (−)-gallocatechin 3-*O*-gallate (GCG), and (−)-epigallocatechin 3-*O*-gallate (EGCG) are effective mushroom tyrosinase inhibitors [41]. These compounds decreased also melanin synthesis in B16 murine melanoma cells [42].

In order to predict which compounds previously detected in *Cistus* extracts are responsible for the tyrosinase inhibition the correlation analysis was performed. Statistically significant correlation between the content of the compounds and tyrosinase inhibitory activity was found for epigallocatechin (R = −0.941; *p* < 0.001), epicatechin (R = −0.955; *p* < 0.001), and myricitrin (R = −0.878; *p* < 0.01) (Table S1). In addition to directly inhibit the enzymatic reaction, EGCG was also shown to decrease melanin synthesis by downregulation of microphthalmia-associated transcription factor (MITF), controlling the expression of various genes related to melanogenesis [43]. Myricetin and myricitrin (myricetin-3-*O*-rhamnoside) were also previously described as weak inhibitors of tyrosinase [44,45]. Tyrosinase inhibitory activity of flavonoids may results from their interaction with free radicals generated at the active site of the enzyme or due to the binding of cooper ions located within the tyrosinase catalytic domains [46,47].

### 3.4. In Vitro Cytotoxicity Against Human Skin Cancer and Noncancerous Skin Cells

Skin protecting properties of cosmetic ingredients may be related not only with their antioxidant and anti-hyperpigmentation activities but could also help to chemoprevention of non-melanoma and melanoma skin cancers. While non-melanoma skin cancers are more numerous, melanoma remains the most challenging disease as it might be fatal if not diagnosed and treated at the early stages. The number of patients diagnosed with this type of cancer increases each year. Therefore, it is necessary to explore new therapeutic and chemopreventive strategies for melanoma management [48]. Cancer chemoprevention involves chronic administration of synthetic or natural agent to suppress the process of cancer initiation, promotion and progression. Including cancer preventing compounds in cosmetics may increase their effectiveness due to the regular and direct application on the skin surface. One major group of phytochemicals that possess such activity are polyphenolic compounds. Among them flavonoids are the most promising agents as they are able to decrease the amount of ROS, reduce the level of DNA damage and mutations and thereby prevents genomic instability and cancer initiation [49]. Currently described melanoma chemopreventive agents of natural origin, effective in topical application, include curcumin, resveratrol, silymarin, and EGCG [50]. 

Among other *Cistus* species only hexane extract from *C. monspeliensis* was shown to possess significant cytotoxic activity against human malignant melanoma cell line A375, with IC_50_ = 52.44 ± 3.69 mg/mL following 72 h treatment [51]. The influence of *C. incanus* and *C. ladanifer* on the skin cancer has not been previously described in the literature. In order to evaluate the potential application of *C. incanus* and *C. ladanifer* extracts in chemoprevention of skin cancer the extracts were assessed for their in vitro cytotoxicity against two types of human skin cancer cells: malignant melanoma (A375) and squamous cell carcinoma (SCC-15) (Table 5). All analyzed *Cistus* extracts were significantly cytotoxic against A375 melanoma cells, with *C. incanus* 100% M and 60% M extracts being the most active (IC_50_ = 57.8 µg/mL and 60.4 µg/mL, respectively). Obtained IC_50_ values following 48 h treatment were much lower than the results obtained for *C. monspeliensis* extract [52], indicating high anticancer potential of *Cistus* extracts obtained by described methodology. The extracts were also analyzed for their cytotoxicity against HaCaT keratinocytes, a spontaneously transformed immortal cell line from adult human skin, widely used in scientific research [53]. The cytotoxicity of all tested extracts against these noncancerous cells was significantly lower than against melanoma, indicating the safety of their potential topical application in melanoma preventing ointments. The cytotoxicity of *Cistus* extracts against other types of cancer cells was previously studied by several research group. Polyphenol rich extract from *C. incanus* reduced viability of human breast (MCF-7) and colon (LOVO) cancer cell lines by 10–30% in the concentration range 1–75 µg/mL but did not influence the viability of noncancerous hamster fibroblast culture (V79) [28]. Aqueous extracts from *C. ladanifer* leaves, also rich in polyphenolic compounds (gallic acid, ellagic acid, and their derivatives) inhibited the proliferation of human M220 pancreatic cancer cells and MCF7/HER2 and JIMT-1 breast cancer cells. Aqueous, ethanol, and acetone:water extracts do not affect the viability of human noncancerous cells such as fibroblasts. Also, HS-766T and M186 pancreatic cancer cells, SKBr3 breast cancer cells and HT29 colon cancer cells showed to be resistant to aqueous *C. ladanifer* extracts [30,31,52]. 

The significant cytotoxic potential of *C. incanus* extracts against melanoma cells might be explained by the high content of phytochemicals previously described to be effective in the chemoprevention and treatment of melanoma, such as EGCG [54,55]. However, the juxtaposition of the phytochemical content of the extracts and cytotoxic activity against A375 cells showed that there is no correlation between the observed cytotoxic activity and the content of major polyphenolic compounds (Table S1). Anticancer activity of polyphenolics, especially catechin derivatives, often results from their synergistic action [56]. For example, the combination of epicatechin and EGCG synergistically inhibited growth of human colon cancer cell line HT-29 [57] and the mixture of EGCG with various green tea catechins decrease proliferation of human cervical cancer cells in vitro [58].

The cytotoxic activity of *Cistus* extracts was also correlated with the tyrosinase inhibitory activity, showing that for most of the extracts cytotoxic effect strongly correlated with tyrosinase inhibitory potential. There was no correlation observed between the cytotoxic activity and the antioxidant potential of analyzed extracts (Table S2, Figures S1 and S2). 

### 3.5. In Vitro Sun Protection Factor (SPF) of Cistus Extracts

UV radiation (UVR) is one of the most harmful environmental factors influencing the physiology of the skin. Exposure to UVR is the major risk factor of skin cancer, it is also responsible for hyperpigmentation disorders and the premature skin aging, known as photoaging [39,59,60]. For that reason UV-protecting ingredients are key components of everyday skin care products. The efficacy of the UV filters is specified by the sun protection factor (SPF), defined as the UV energy required for producing a minimal erythema dose (MED) on protected skin, divided by the UV energy required producing a MED on unprotected skin. MED is defined as the lowest time interval or dosage of UV irradiation sufficient for producing a minimal, visible erythema on unprotected skin [61]. Several plant extracts were already described as natural UV filters, suggesting their valuable contribution to the overall SPF of skin protecting cosmetics [62]. High content of polyphenolic compounds indicated that *Cistus* extracts may also possess UVR absorbing activity. To establish the SPF of *Cistus* extracts the in vitro method applying Mansur equation was used [25]. All of the analyzed *Cistus* extracts at 100 µg/mL showed comparable SPF, ranging from 3.33 to 4.37 (Table 6). The highest SPF was measured for the extracts prepared by Soxhlet extraction in 100% methanol. The potential application of *Cistus* extracts in UV-protecting cosmetics is also supported by the work of Attaguile and co-workers (2000), showing that aqueous extracts from *C. incanus* aerial parts directly protected DNA from UVR-mediated oxidative damage [63].

## 4. Conclusions

This study compares for the first time the phytochemical composition and biological properties of *C. incanus* and *C. ladanifer* extracts, supporting their application as multifunctional active ingredients in cosmetics protecting the skin from the harmful external factors. Conducted study showed that the extracts from both species possess significant antioxidant properties, tyrosinase inhibitory activity, and UV protecting potential. All analyzed extracts were also cytotoxic for human malignant melanoma cells in vitro and did not impair the growth of noncancerous skin cells (keratinocytes). Among analyzed extracts the most interesting properties, important for the skin protection, were detected for *C. incanus* extracts prepared in 60% (*v*/*v*) methanol, using magnetic stirrer or Soxhlet apparatus extraction. These extracts contained the highest amount of phytochemicals (331.82–347.27 mg GAE/g DW) as well as the greatest variety of compounds. *C. incanus* extracts were also described for the first time as effective tyrosinase inhibitors, decreasing dopaquinone formation from l-DOPA by 30–70% at 100 µg/mL. The correlation analysis helped to indicate the ingredients responsible for tyrosinase inhibition—epicatechin, EGCG, and myricitrin. Tyrosinase inhibitory activity is particularly interesting for cosmetic application due to the increasing problem of hyperpigmentation disorders and growing consumer demand for safe and effective skin lightening cosmetics [38,39]. *C. incanus* extracts were also the most cytotoxic for human melanoma cells in vitro, with IC_50_ values between 57.80 and 109.41 µg/mL. The anticancer activity of these extracts results more likely from the synergistic action of several phytochemical ingredients. Anti-hyperpigmentation and anti-melanoma potential of *C. incanus* extracts is also complemented by their natural sun protecting activity (SPF 3.42–3.77 at 100 µg/mL), as excessive UVR exposure is a major cause of increased melanin synthesis and skin cancerogenesis [1]. 

To summarize, the data obtained from in vitro experiments, presented in this manuscript strongly indicate that *C. incanus* and *C. ladanifer* extracts may be effective active ingredients for the cosmetic industry. However, the antioxidant, skin lightening and UV-protecting properties of *C. incanus* and *C. ladanifer* extracts as components of cosmetic formulations require further investigation using 3D in vitro skin equivalents or in vivo models.

## Figures and Tables

**Table 1:** Normalized product function used in the calculation of SPF. Values adapted from the work of Sayre and co-workers [26].

Wavelenght (λ, nm)	EE *×* I (Normalized)
290	0.0150
295	0.0817
300	0.2874
305	0.3278
310	0.1864
315	0.0839
320	0.0180
**Total**	**1**

EE—erythremal effect spectrum, I—solar intensity spectrum.

**Table 2:** The content of total phenolics, flavonoids and DPPH scavenging activity in *C. incanus* and *C. ladanifer* extracts.

		Total Phenolic Content (mg GAE/g DW)	Total Flavonoid Content (mg QuE/g DW)	DPPH Scavenging (IC_50_, µg/mL)
*C. incanus*	60% M	331.82 ± 13.39 ^b,c^	44.76 ± 0.45 ^a^	4.05 ± 0.43 ^a,b^
	60% SOX	347.27 ± 17.03 ^c^	53.76 ± 0.89 ^b^	3.81 ± 0.36 ^a^
	100% M	297.71 ± 13.77 ^a,b^	44.77 ± 1.64 ^a^	4.76 ± 0.18 ^a,b,c^
	100% SOX	269.28 ± 23.62 ^a^	50.85 ± 0.54 ^b^	16.75 ± 0.47
*C. ladanifer*	60% M	267.58 ± 3.78 ^a^	32.35 ± 0.94	5.49 ± 0.48 ^b,c^
	60% SOX	301.82 ± 10.91 ^a,b^	42.35 ± 1.32 ^a^	4.08 ± 0.31 ^a,b^
	100% M	142.81 ± 6.30	27.91 ± 1.02	10.20 ± 1.37
	100% SOX	191.18 ± 6.79	37.54 ± 1.88	5.88 ± 0.26 ^c^

Each value represents mean ± SD (*n* = 3). Means not sharing the same letter (a–c) are significantly different at *p* < 0.05.

**Table 3:**
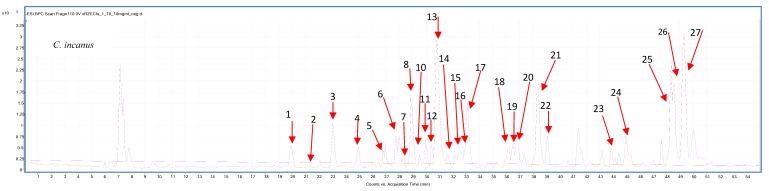
Results of the qualitative LC-MS study of *C. incanus* and *C. ladanifer* extracts (delta—the calculated molecular weight error, DBE—double bond equivalent, deriv—derivative, Tr—traces).

No	Ion (+/−)	Rt (min)	Molecular Formula	m/z Calculated	m/z Experimental	Delta(mmu)	RDB	MS/MS Fragments	Proposed Compound	C. incanus	C. ladanifer	References
1	[M − H]^−^	19.9	C_7_H_6_O_5_	169.0142	169.0151	−5.02	5	125, 97	Gallic acid	+++	+++	[12,37,38]
2	[M − H]^−^	21.2	C_30_H_26_O_14_	609.1250	609.1251	−0.2	18	441, 423, 303	Epigallocatechin dimer	+++	+++	[12,38]
3	[M − H]^−^	23.0	C_15_H_14_O_7_	305.0667	305.0658	2.86	9	361, 219, 167	Epigallocatechin	+++	+	[12,37,38]
4	[M − H]^−^	24.9	C_15_H_14_O_7_	305.0667	305.0665	0.58	9	221, 179, 166,	Gallocatechin	+	Tr	[12]
	[M − H]^−^	25.5	C_12_H_14_O_8_	285.0616	285.0589	9.41	6	153, 108	Uralenneoside	Tr	++	[15]
5	[M − H]^−^	26.8	C_15_H_14_O_6_	289.0718	289.0692	8.83	9	247, 203, 137	Epicatechin	++	Tr	[12]
6	[M − H]^−^	27.7	C_16_H_22_O_8_	341.1242	341.1246	−1.19	6	179	Caffeoyl-glucose	++	+	[12]
7	[M − H]^−^	28.3	C_15_H_14_O_6_	289.0718	289.0696	7.45	9	-	Catechin	Tr	-	[12,38]
8	[M − H]^−^	28.8	C_21_H_20_O_13_	479.0831	479.0859	−5.8	12	316, 271	Myricetin hexoside	+++	+	[12]
9	[M − H]^−^	29.2	C_20_H_15_O_12_	447.0558	447.0597	−6.25	13	300, 175	Ducheside A	+	+	[15,37]
10	[M − H]^−^	29.5	C_27_H_29_O_16_	609.1461	609.1490	−4.74	13	301, 151	Rutoside	+	+	[12,37]
11	[M − H]^−^	29.9	C_23_H_26_O_12_	493.1351	493.1385	−6.78	11	313, 179	Kaempferol dimethylether glucoside	++	+	[15,37]
12	[M − H]^−^	30.3	C_20_H_18_O_12_	449.0725	449.0703	5.00	12	316	Myricetin pentoside	++	+	
13	[M − H]^−^	30.7	C_21_H_20_O_12_	463.0882	463.0877	1.08	12	316, 271	Myricitrin	+++	+++	[12]
	[M − H]^−^	31.5	C_16_H_24_O_7_	327.1449	327.1428	6.48	5	228, 165	Betuloside	Tr	++	[15]
14	[M − H]^−^	32.1	C_20_H_15_O_12_	447.0933	447.0937	−0.93	12	284, 201	Kaempferol glycoside	+	+	[12,37]
15	[M − H]^−^	32.5	C_20_H_18_O_11_	433.0776	433.0772	1.00	12	345, 300, 151	Quercetin pentoside	++	+	[12]
16	[M − H]^−^	32.7	C_20_H_15_O_12_	447.0933	447.0927	1.31	12	403, 284, 174	Kaempferol glycoside	+	Tr	[12,37]
17	[M − H]^−^	33.01	C_21_H_20_O_11_	447.0933	447.0956	−5.17	12	393, 301, 179	Quercetrine	++	++	[12]
18	[M − H]^−^	36.1	C_23_H_20_O_9_	439.1035	439.1037	−0.55	14	421, 409, 393, 287, 260	Unknown compound	+	-	
19	[M − H]^−^	36.4	C_14_H_6_O_8_	300.999	300.9986	1.29	12	283, 229	Ellagic acid	Tr	Tr	[37]
20	[M − H]^−^	36.8	C_15_H_10_O_8_	317.0303	317.0305	−0.66	11	255, 179	Myricetin	+++	Tr	[12]
21	[M − H]^−^	38.4	C_30_H_26_O_13_	593.1301	593.1340	1.79	18	447, 285, 223	Kaempferol diglycoside	+++	+++	[15,37]
22	[M − H]^−^	39.3	C_30_H_26_O_13_	593.1301	593.1315	−2.42	18	447, 285	Kaempferol diglycoside	++	++	[15,37]
23	[M − H]^−^	43.9	C_39_H_32_O_15_	739.1668	739.1677	−1.16	24	593, 453, 285	Kaempferol dicoumaroyl glucose	+++	+++	[12]
24	[M − H]^−^	44.8	C_39_H_32_O_15_	739.1668	739.1660	1.14	24	593, 453, 285	Kaempferol dicoumaroyl glucose	+++	+++	[12]
25	[M − H]^−^	48.3	C_17_H_26_O_4_	293.1758	293.1751	2.49	5	249, 236, 221	Unknown compound	++	++	
26	[M − H]^−^	48.6	C_16_H_11_O_5_	283.0612	283.0589	8.09	11	268, 257	Apigenin methylether	++	++	[15,37]
27	[M − H]^−^	49.3	C_17_H_13_O_6_	313.0718	313.0722	−1.4	11	298, 283, 255	Kaempferol dimethylether	+++	+++	[15,37]

The content of identified compounds was correlated with previously showed antioxidant activity of *Cistus* extracts. This analysis revealed that the concentration of epigallocatechin (R = 0.902; *p* < 0.01) and epicatechin (R = 0.845; *p* < 0.01) in the extracts strongly correlates with their DPPH scavenging potential (Table S1).

**Table 4:** Quantitative LC/MS analysis of *C. incanus* and *C. ladanifer* extracts.

	*C. incanus*				*C. ladanifer*			
	60% M	60% SOX	100% M	100% SOX	60% M	60% SOX	100% M	100% SOX
Ellagic acid	41.39 ^a^ ± 0.83	55.15 ^b^ ± 1.10	88.66 ± 2.50	656.37 ± 5.20	44.84 ^a,b^ ± 0.64	609.89 ± 6.10	264.36 ± 8.80	199.55 ± 1.20
Gallic acid	278.86 ^c^ ± 10.00	298.49 ^c^ ± 17.00	283.39 ^c^ ± 6.30	772.90 ± 11.00	301.98 ^c^ ± 12.00	-	614.18 ± 38.00	541.61 ± 16.00
Quercetrine	687.27 ± 22.00	547.40 ±34.00	1275.09 ± 75.00	2473.45 ± 64.00	-	46.63 ± 0.25	-	-
Epicatechin	100.49 ^d^ ± 8.60	67.34 ^e^ ± 3.40	104.07 ^d^ ± 6.20	59.93 ^e^ ± 0.99	trace	-	trace	trace
Gallocatechin	3.06 ± 0.17	115.35 ± 5.20	-	-	-	-	-	-
Epigallocatechin	891.87 ^f^ ± 4.90	591.42 ^f^ ± 22.00	7271.59 ± 300.00	1384.63 ± 34.0	164.33 ^g^ ± 4.40	3.22 ± 0.15	191.46 ^g^ ± 6.20	38.93 ± 1.10
Epigallocatechin dimer	298.96 ± 7.00	83.08 ± 3.60	67.44 ± 1.50	25.76 ± 0.52	17.56 ^h^ ± 1.10	2.22 ^I,j^ ± 0.16	9.50 ^h,I^ ± 0.27	0.48 ^j^ ± 0.02
Rutoside	61.02 ± 3.90	40.17 ± 1.20	20.28 ± 0.79	171.54 ± 3.10	5.40 ^k^ ± 0.32	1.44 ^k^ ± 0.08	4.09 ^k^ ± 0.25	2.66 ^k^ ± 0.15
Kaempferol glucoside	103.62 ^l^ ± 3.20	112.07 ^l^ ± 7.70	211.11 ^n^ ± 7.30	638.59 ± 51.0	84.38 ^l,m^ ± 1.00	44.44 ^m^ ± 2.00	204.48 ^n^ ± 6.20	211.27 ^n^ ± 4.80
Kaempferol diglycoside	246.10 ±7.10	414.49 ± 8.80	625.44 ± 14.00	5889.89 ± 9.80	290.66 ± 7.30	522.16 ± 13.00	851.36 ± 22.00	777.14 ± 9.30
Kaempferol diglycoside	79.38 ^o^ ± 3.30	123.73 ^o,p^ ± 7.50	244.77 ± 61.00	442.52 ^r^ ± 11.00	90.24 ^o^ ± 1.50	168.82 ^p^ ± 6.40	379.65 ^r^ ± 9.20	700.03 ± 12.00
Myricetin	73.43 ^s^ ± 0.18	236.33 ± 5.60	38.48 ^s^ ± 1.00	1076.44 ± 39.00	-	-	-	343.53 ±8.80
Myricitine pentoside	621.04 ^t^ ± 13.00	440.80 ± 9.50	636.38 ^t^ ± 11.00	1808.26 ± 67.00	-	-	-	-
Myricitrin	1310.94 ± 74.00	1079.80 ± 66.00	1795.36 ± 43.00	604.22 ± 15.00	62.89 ^u^ ± 2.60	23.00 ^u^ ± 0.81	41.04 ^u^ ± 1.80	23.13 ^u^ ± 0.69
Quercetin pentoside	246.01 ± 6.70	136.85 ± 4.10	427.72 ± 17.00	860.18 ± 9.20	trace	-	-	-

Each value represents mean content of the compound (µg/g DW) ± SD (*n* = 3), means not sharing the same letter (a–p, r–u) are significantly different at *p* < 0.05 probability level in each column.

**Table 5:** In vitro cytotoxicity of *C. incanus* and *C. ladanifer* extracts against human malignant melanoma (A375), human scquamous cell carcinoma (SCC-15), and non-cancerous human keratinocyte (HaCaT) cell lines; each value represents mean IC_50_ (µg/mL) from three experiments.

	IC_50_ (µg/mL)							
	*C. incanus*				*C. ladanifer*			
	60% M	60% SOX	100% M	100% SOX	60% M	60% SOX	100% M	100% SOX
A375	60.41	84.62	57.80	109.41	95.30	138.61	199.01	164.91
SCC-15	>500	>500	383.61	>500	>500	>500	>500	>500
HaCaT	319.71	>500	343.40	>500	>500	>500	>500	>500

**Table 6:** Determination of the sun protection factor (SPF) of *C. incanus* and *C. ladanifer* extracts.

	Sun Protection Factor (100 µg/mL Extract)	
	*C. incanus*	*C. ladanifer*
60% M	3.42 ± 0.13 ^a^	3.33 ± 0.15 ^a^
60% SOX	3.77 ± 0.28 ^a,b^	3.50 ± 0.52 ^a^
100% M	3.64 ± 0.08 ^a,b^	3.71 ± 0.30 ^a,b^
100% SOX	4.13 ± 0.19 ^a,b^	4.37 ± 0.42 ^b^

Each value represents mean ± SD (*n* = 3). Means not sharing the same letter (a and b) are significantly different at *p* < 0.05.

## Supplementary Materials

The following are available online at https://www.mdpi.com/2076-3921/9/3/202/s1; Figure S1: Dendrograms showing similarities between the tested extracts depending on DPPH (A), tyrosinase activity (B), cytotoxic activity (C) and SPF results (D). Figure S2: Graphs showing the content of total phenolics, flavonoids, DPPH scavenging activity in *C. incanus* and *C. ladanifer* extracts and determination of the Sun Protection Factor (SPF) of *C. incanus* and *C. ladanifer* extracts; each value represents mean ± SD (*n* = 3). Means not sharing the same letter are significantly different at *p* < 0.05. Table S1: Correlation between the content of phytochemical compound and biological activity of *Cistus* extracts. Table S2. Correlation between tyrosinase inhibitory activity and antioxidant activity of *Cistus* extracts and cytotoxicity against A375 melanoma cells.
