# Supplementary material for: Characterization of Cistus × incanus L. and Cistus ladanifer L. Extracts as Potential Multifunctional Antioxidant Ingredients for Skin Protecting Cosmetics
Source: Antioxidants (Basel). 2020 Mar 1;9(3):202. doi: 10.3390/antiox9030202 (PMC7139296; doi:10.3390/antiox9030202)
Supplement: Supplementary file 1 [file antioxidants-09-00202-s001.pdf]

Supplementary Materials:

Table S1. Correlation between the content of phytochemical compound and biological activity of *Cistus* extracts**.**

	ellagic acid	***gallic acid***	***quercetrine***	***kaempferol glucoside***	***epigallocatechin***	***gallocatechin***	***epicatechin***	***rutoside***	***epigallocatechin dimer***	***kaempferol diglycoside***	***kaempferol diglycoside***	***myricetin***	***myrecitine pentoside***	***myricitrin***	***quercetin pentoside***
Antioxidant activity	-.174	.182	.575	.432	.902**	-.199	.845**	.218	-.120	.321	.297	.271	.456	.575	.599
Tyrosinase inhibitory activity	.245	.232	-.490	-.064	-.941***	-.127	-.955***	-.065	-.256	.207	.333	.0660	-.365	-.878**	-.495
Cytotoxicity against A375	-.130	.513	.460	.533	.253	.478	.204	.469	-.244	.564	.329	.512	.395	.252	.429

** $p < 0.01$, *** $p < 0.001$.

Table S2. Correlation between tyrosinase inhibitory activity and antioxidant activity of *Cistus* extracts and cytotoxicity against A375 melanoma cells.

	C. incanus **60% M**	***C. ladanifer*** **60% M**	***C. incanus*** **60% SOX**	***C. ladanifer*** **60% SOX**	***C. incanus*** **100% M**	***C. ladanifer*** **100% M**	***C. incanus*** **100% SOX**	***C. ladanifer*** **100% SOX**
	Cytotoxicity against A375							
Antioxidant activity	-.730	-.762	-.396	-.519	-.280	-.184	-.209	-.547
Tyrosinase inhibitory activity	.955*	.972**	.976**	.893*	.957*	.489	.962**	.723
	Antioxidant activity							
Tyrosinase inhibitory activity	-.807	-.637	-.609	-.699	-.538	-.591	-.354	-.578

* $p < 0.05$, ** $p < 0.01$.

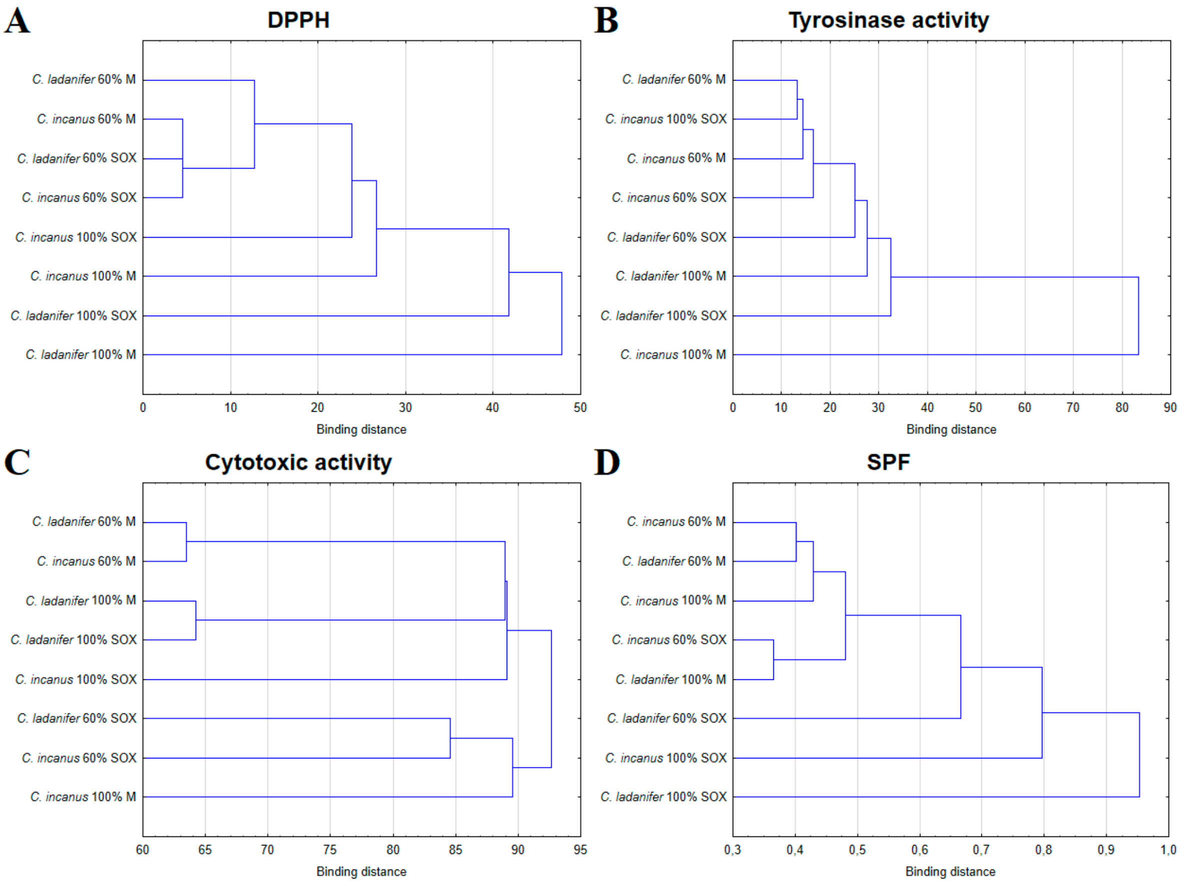

Figure S1. Dendrograms showing similarities between the tested extracts depending on DPPH (**A**), tyrosinase activity (**B**), cytotoxic activity (**C**) and SPF results (**D**).

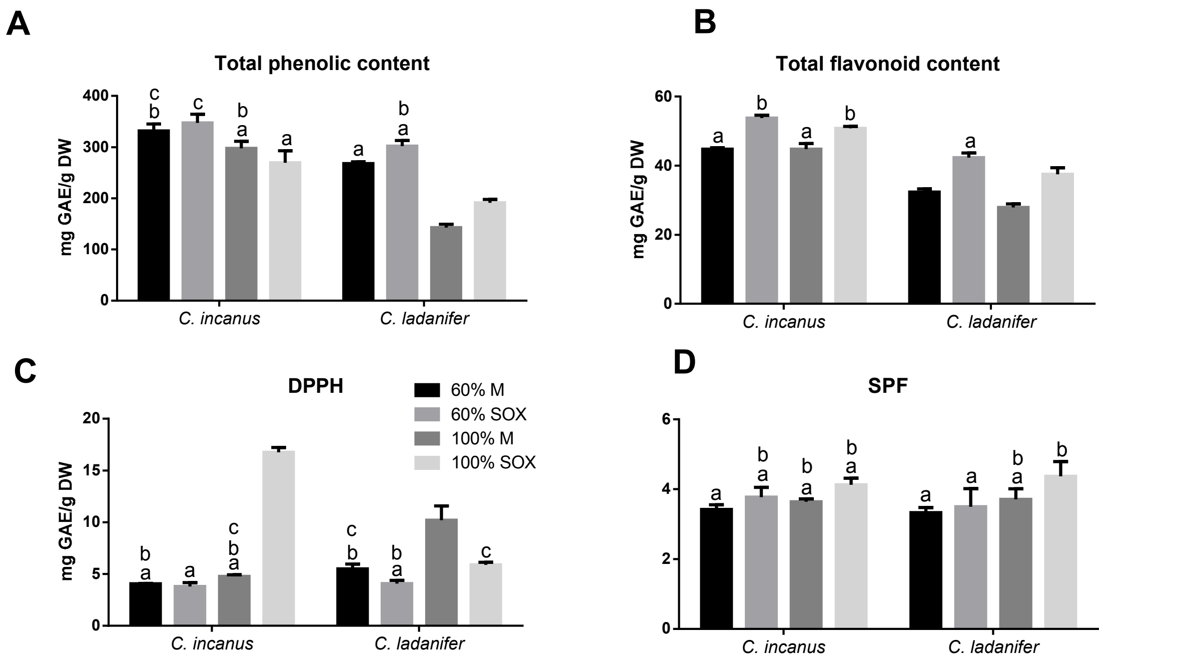

Figure S2. Graphs showing the content of total phenolics, flavonoids, DPPH scavenging activity in *C. incanus* and *C. ladanifer* extracts and determination of the Sun Protection Factor (SPF) of *C. incanus* and *C. ladanifer* extracts; each value represents mean ± SD ($n = 3$). Means not sharing the same letter are significantly different at $p < 0.05$.
